# MuTATE: an interpretable multi-endpoint machine learning framework for automated molecular subtyping in cancer

**DOI:** 10.1038/s44401-025-00025-4

**Published:** 2025-07-03

**Authors:** Sarah G. Ayton, Martina Pavlicova, Carla Daniela Robles-Espinoza, Rita Q. Fuentes-Aguilar, Debora Garza-Hernandez, Emmanuel Martínez-Ledesma, Jose Gerardo Tamez-Peña, Mario R. Garcia-Pompermayer, Víctor Treviño

**Affiliations:** 1 Tecnologico de Monterrey, Escuela de Medicina y Ciencias de la Salud, Monterrey, Mexico; 2 Tecnologico de Monterrey, Escuela de Ingeniería y Ciencias, Monterrey, Mexico; 3 Columbia University, Mailman School of Public Health, New York, NY USA; 4 Cornell University, Weill Cornell Medicine, New York, NY USA; 5 Universidad Nacional Autónoma de México, Laboratorio Internacional de Investigación sobre el Genoma Humano, Campus Juriquilla, Santiago de Querétaro, Querétaro, Mexico; 6 Wellcome Sanger Institute, Wellcome Trust Genome Campus, Cambridge, UK; 7 Tecnologico de Monterrey, The Institute of Advanced Materials and Sustainable Manufacturing, Guadalajara, Mexico; 8 Tecnologico de Monterrey, The Institute for Obesity Research, Monterrey, Mexico; 9 Tecnologico de Monterrey, Hospital San Jose, TecSalud, Monterrey, Mexico; 10 Tecnologico de Monterrey, oriGen Project, Monterrey, Mexico

**Keywords:** Cancer, Biomarkers, Health care, Molecular medicine, Oncology, Risk factors, Applied mathematics, Computational science, Computer science, Computational biology and bioinformatics, Machine learning

## Abstract

Effective and interpretable molecular subtyping is critical for cancer risk stratification and treatment, yet existing methods face key limitations. Traditional models cannot jointly model multiple clinical endpoints, limiting prognostic utility, while machine learning (ML) approaches often lack transparency. We developed MuTATE, an automated, interpretable decision-tree framework powered by ML that improves subtyping accuracy and enables multi-endpoint risk stratification. MuTATE was evaluated using 18,400 simulations and 682 patient biopsies from three TCGA cancers: lower-grade glioma (LGG), endometrial carcinoma (EC), and gastric adenocarcinoma (GA). Compared to established clinical models, MuTATE improved accuracy, interpretability, and biomarker discovery, and reclassified risk groups. In LGG, MuTATE reassigned 13% of “low-risk” IDH-1p19q cases into higher-risk subtypes, and 19% of “high-risk” IDH wild-type cases were reassigned to higher-risk categories. In GA, MuTATE refined the “intermediate-risk” genomically stable group into a higher-risk ARID1A wild-type subtype. In EC, 72% of “intermediate-risk” MSI/MLH1 cases were reassigned to the highest-risk category. These findings demonstrate MuTATE’s potential to reduce diagnostic bias, improve risk stratification, and support scalable integration of multi-endpoint ML into precision oncology workflows.

## Introduction

Achieving comprehensive and interpretable molecular subtyping for precise risk stratification and targeted treatments in cancer is a critical challenge in clinical practice and precision medicine^1–4^. Clinical, expert-derived, decision-tree models for subtyping cancers, such as those used in lower-grade glioma (LGG), gastric adenocarcinoma (GA), and endometrial carcinoma (EC)^5–7^, have played a key role in patient risk stratification based on molecular features^8–12^. However, these models require extensive and time-consuming domain expertise and they lack formal optimization, raising questions about their efficacy in capturing the best possible classification outcomes.

Cancer presents as a complex and diverse disease, with molecular alterations disrupting multiple pathways and influencing disease progression, treatment response, and patient survival. This complexity contributes to the staggering 19.3 million new cases and 10 million deaths observed in 2020, with a projected 47% rise in cancer burden over the next two decades^4^. Despite the recent integration of molecular biomarkers with histology in models such as the WHO glioma classification^13,14^, (which centers on optimizing treatment outcomes by molecular subtype)^15–21^, existing subtyping methods fall short in providing comprehensive characterization across multiple clinical endpoints, limiting their prognostic value^22^.

Advanced machine learning (ML) algorithms, while powerful, struggle with overfitting, limited interpretability, and potential bias^23–30^, especially when applied to multi-endpoint diseases. Many state-of-the-art methods, though accurate, sacrifice interpretability for performance, making them less suitable for clinical use where transparency and explainability are critical^22,31,32^. A superior risk classification model for clinical use not only improves predictive accuracy but also offers enhanced clinical interpretability, efficiently integrating multiple endpoints to guide decision-making and provide actionable insights into patient outcomes. Attempts to adapt decision trees^33^ for clinically interpretable ML models have not addressed the multi-endpoint challenge in complex molecular data (e.g., allowing only continuous endpoints or managing multiple endpoint variable types in separate models)^23,34–36^. Non-interpretable ML models, such as neural networks or random forests, while achieving high accuracy, offer little transparency in decision-making processes, making them less useful in clinical contexts where interpretability is crucial. While ensemble methods that combine multiple single-endpoint trees improve accuracy, they compromise explainability, masking key insights into disease subtypes and treatment strategies^25–30^.

To address the constraints of ML, expert clinicians have resorted to constructing manually derived decision tree architectures (expert trees), to explain relationships between molecular signatures and clinical outcomes^5–7^. Expert trees, while more interpretable, are labor-intensive, potentially biased, and predominantly built for European-descendant populations, limiting their broader applicability^37–43^, To address these challenges, there is an urgent need for automated, multi-endpoint subtyping methods that enhance interpretability, reduce bias, and support equitable precision medicine across diverse populations^22,44^.

To overcome these challenges, Multi-Target Automated Tree Engine (MuTATE), an ML-enabled algorithm was developed to automate the creation of clinically interpretable decision-tree models for complex, multi-endpoint diseases like cancer^45^. MuTATE addresses the limitations of traditional models by optimizing molecular subtyping without the need for extensive manual input or domain expertise, thereby reducing bias and improving explainability. In previous work, we introduced MuTATE^19,45^, and here, we present the first cohort study applying it to real-world cancer data. Our study compares MuTATE’s performance against established expert tree models and advanced ML in three cancers: LGG, EC, and GA^5–7^. We focus on these three cancer types—LGG, EC, and GA—as they are the only publicly available cohorts from TCGA with manually-constructed clinical decision tree models, providing a direct framework for comparison with MuTATE.

MuTATE outperformed previous models in identifying novel molecular signatures, improving multi-endpoint subtyping accuracy, and enhancing clinical explainability. By automating molecular subtyping with interpretable models, MuTATE democratizes biomarker discovery, enabling its application across diverse datasets, populations, and resource-limited settings. This flexibility ensures its relevance in advancing equitable precision medicine and optimizing patient outcomes.

## Results

Demographic and clinical characteristics were significantly different between cohorts (Supplementary Data 2). Supplementary Data 2 offers detailed clinical and demographic breakdowns that reveal key differences in age, race, and survival across cohorts—context that anchors later stratification findings. For example, patients tended to be younger (avg. 42.7 years, *p* < 0.001), predominantly white (*N* = 260, 95.2%, *p* < 0.001) and had the highest rates of remaining with tumor (*N* = 139, 58.6%, *p* < 0.001) in the LGG cohort, more male patients (*N* = 111, 65.7%, *p* < 0.001) with the shortest overall survival (avg. 467.0 days, *p* < 0.001) and tumor-free survival (avg. 381.5 days, *p* < 0.001) in the GA cohort, and more diverse (non-white *N* = 53, 22.5%, *p* < 0.001) patients with the longest overall survival (avg. 1030.5 days, *p* < 0.001) and progression-free survival (avg. 929.1 days, *p* < 0.001) in the EC cohort.

### MuTATE outperforms CART in simulations

**Fig. 1:**
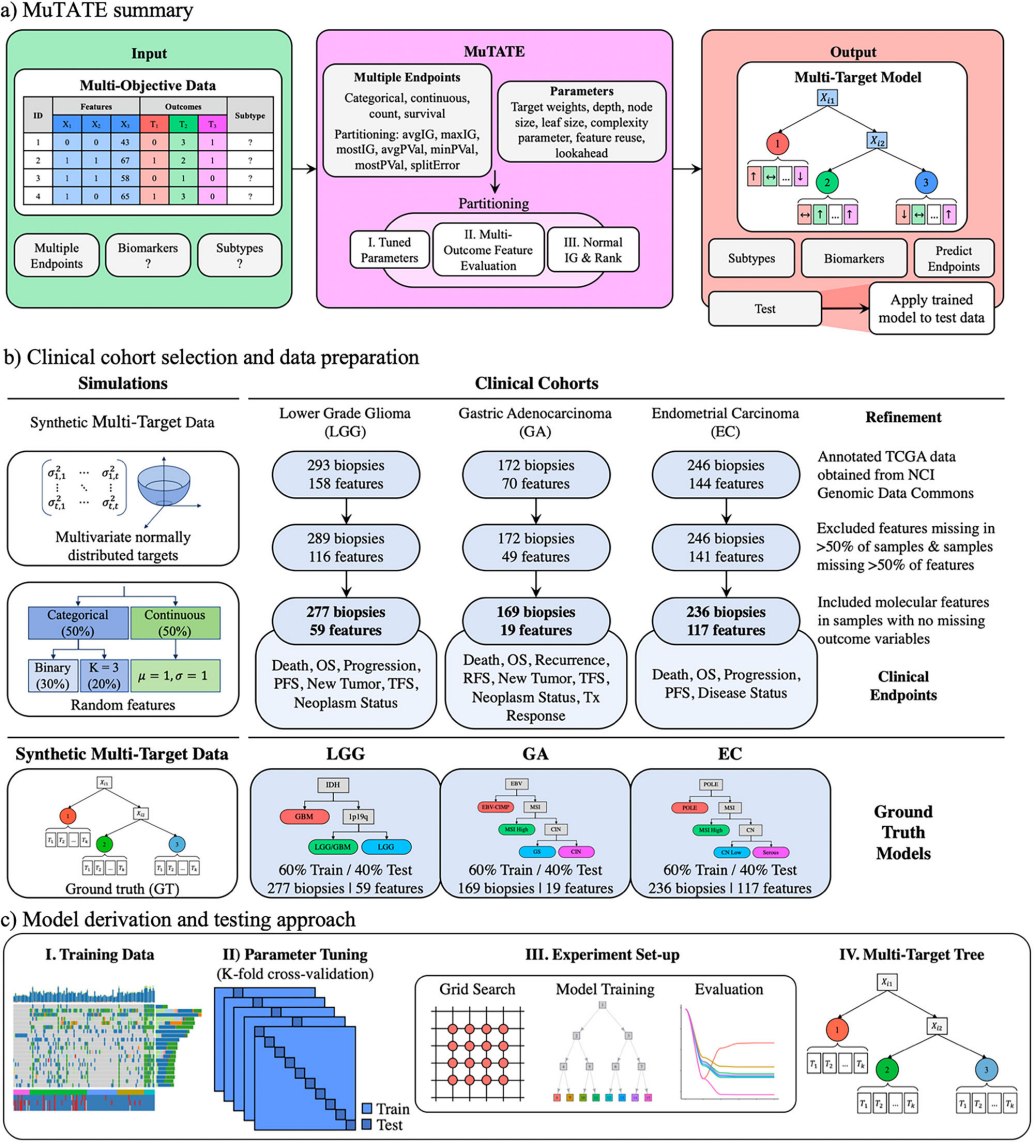
A complex multi-target tree-structured approach enables MuTATE for automated prognostic biomarker and subtype discovery. **a** MuTATE enables explainable multi-endpoint ML by evaluating features across clinical endpoints^45^. Partitions are based on information gained (IG) using highest average multi-target IG (avgIG), highest IG in any target (maxIG), meaningful IG in the most targets (mostIG), lowest average p-value of statistically significant IG (avgPVal), lowest p-value, weighted by number of targets with significant IG (minPVal), significant IG in the most targets (mostPVal), or subtree lookahead (splitError). Trees predict endpoints and identify biomarkers and subtypes. **b** Synthetic multi-target data were generated using a positive definite covariance matrix of targets using a correlation structure (mean $\mu$ = 1, SD $\sigma$ = 1). Features were generated and sampled with replacement for ground truth (GT) definition, targets were divided into leaf quantiles and randomly assigned, resulting in multi-target tree-structured data with a known GT. Clinical cohorts with established expert trees were obtained from TCGA from the NCI Genomic Data Portal. 682 biopsies from three cohorts of 711 patients were included. **c** In simulations, synthetic data and GT are divided into train/test sets (60/40 data split), and grid search assesses model parameters for model test error, TDR, FDR in 18,400 synthetic datasets. Clinical cohorts were divided into train/test sets (60/40 data split), training sets underwent parameter tuning, model performance was captured. Tuned parameters used in trained models were applied to the full cohorts. Final trees were assessed for prognostic significance of partitions, biomarkers, and subtypes. See Figs. S1-6.

**Fig. 2:**
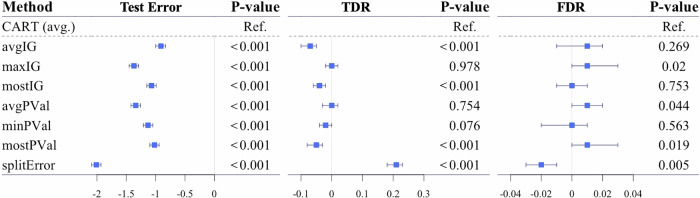
MuTATE is an explainable ML algorithm for accurate multi-target structural modeling. Multivariable analyses assessing method and model performance in 18,400 synthetic multi-target dataset simulations (adjusted for simulated sample size, number of targets, number of features, inter-target correlation, model depth, and run ID). Boxplots represent regression coefficient distributions across 18,400 simulations. Points indicate mean coefficient estimates with 95% CI. Positive coefficients reflect improved performance relative to CART (e.g., higher TDR), while negative coefficients reflect lower error or FDR. Whiskers represent interquartile range, and overlaid points display mean coefficients with 95% confidence intervals, derived from multivariable regression adjusting for simulation conditions.

In the evaluation of 18,400 simulations, MuTATE models consistently demonstrated superior performance over CART, yielding significantly improved error, as well as improved true and false discovery rates in multivariable analyses. (Fig. 2, Figs. S1–2, Supplementary Data 1). In each simulation, 100 synthetic multi-target GT trees were constructed, synthetic sets were divided into train and test sets (60/40 data split), and grid search assessed MuTATE trees and averaged single-target CART models for test error, true discovery rate (TDR), and false discovery rate (FDR) across model parameters in 18,400 synthetic datasets (Fig. 1b, c, Fig. S2).

Figure 1 provides an overview of the MuTATE framework, including the model architecture (a), data preparation and cohort characteristics (b), and evaluation strategy across simulations and clinical datasets (c). Panel 1a outlines how multi-endpoint inputs are processed to generate interpretable decision trees. Panel 1b contrasts synthetic data generation with real TCGA cohort preprocessing. The left side shows how synthetic datasets were constructed to mimic multi-endpoint complexity, while the right side displays key statistics (e.g., sample size, endpoints, features) for each TCGA cohort. Panel 1c then illustrates the end-to-end evaluation pipeline—from data split and parameter tuning to model testing—clarifying the experimental setup across both synthetic and clinical datasets.

While CART built separate models for each endpoint and was unable to explainably capture the GT, MuTATE accurately identified the GT with improved performance in one interpretable model, highlighting its superior ability to explainably and accurately represent complex data in a clear visual model (Fig. S1). CART had the highest test error (2.97, 95%CI: 2.93–3.01). MuTATE outperformed CART for model depths of two and above. As targets increased, MuTATE FDR dropped from 12.7% (2-target 95%CI: 8.7–16.7%) to 5.7% (5-target 95%CI: 2.7–8.7%), while CART maintained a constant FDR (11.0%, 95%CI: 9.0–12.0%). Sample size and inter-target correlation did not show a clear trend in performance of either method. As features increased, a decline in performance was observed across all methods, including CART. In multivariable logistic regression analysis of simulation performance, adjusting for simulated characteristics, all MuTATE partitioning options showed statistically significantly lower test error compared with CART (*p* < 0.001) (Fig. 2, Supplementary Data 1). MuTATE using splitError partitioning showed statistically significantly higher TDR (p < 0.001), and lower FDR compared with CART (*p* = 0.005), adjusting for simulated characteristics (Supplementary Data 1). MuTATE demonstrated superior explainability in constructing clear and interpretable models that accurately capture the underlying GT in complex data.

### Clinical cohorts: real-world validation of MuTATE

To assess real-world performance, we applied MuTATE to three independent clinical cohorts from TCGA (LGG, EC, and GA), each with corresponding expert-derived decision trees. These represent the only TCGA cohorts with such clinical models, allowing rigorous validation against established clinical subtypes. In cross-validated model selection across three cohorts, MuTATE significantly outperformed CART, highlighting its utility for clinical modeling (Supplementary Data 3). CART did not produce meaningful models on these clinical cohorts, as it failed to identify any partitions or key biomarkers in the datasets, resulting in 0 partitions. This outcome further demonstrates the robustness of MuTATE in stratifying disease heterogeneity and identifying relevant molecular features for precision medicine applications. Clinical endpoints for modeling included overall survival (OS, all), tumor-free survival (TFS, all), progression-free survival (PFS, LGG and GA), recurrence-free survival (RFS, GA and EC), vital status (all), neoplasm status (LGG and GA), new tumor (LGG and GA), recurrence (GA), progression (LGG and EC), and treatment response (GA) (Supplementary Data 2). Clinical cohorts were divided into train and test sets (60/40 data split), training data underwent k-fold cross-validated parameter tuning using grid search, model performance was captured, the best performing ML method was selected for tree construction, and final trees were applied to full cohorts (Fig. 1b, c). TDR and FDR were calculated based on manually constructed expert models as GT. MuTATE outperformed CART in cross-validated parameter tuning, highlighting improved ability to capture data complexity in clinical applications (Supplementary Data 3). Supplementary Data 3 outlines cross-validated performance metrics across all MuTATE partitioning strategies, enabling comparison of statistical heuristics in each cancer type. To identify the best-performing strategies across cohorts, we evaluated all MuTATE partitioning methods. Notably, avgIG performed best in LGG, splitError in GA, and mostPVal in EC—each outperforming CART on all performance metrics (Supplementary Data 3). MuTATE using avgIG (LGG), splitError (GA), and mostPVal (EC) partitioning achieved the highest TDR (LGG: 91%, 95%CI: 82–99%; GA: 42%, 95%CI: 24–59%; EC: 1.00, 95%CI: 1.00–1.00) and lowest test error (LGG: 0.07, 95%CI: 0.06–0.08, GA: 0.13, 95%CI: 0.06–0.20; EC: 0.15, 95%CI: 0.14–0.15) compared with CART, which had the lowest TDR and highest test error. Multi-target trees showed consistent improvement in test error, TDR, and model explainability, automating expert architectures across clinical cohorts.

### Refined molecular subtypes enhance prognostic stratification

**Fig. 3:**
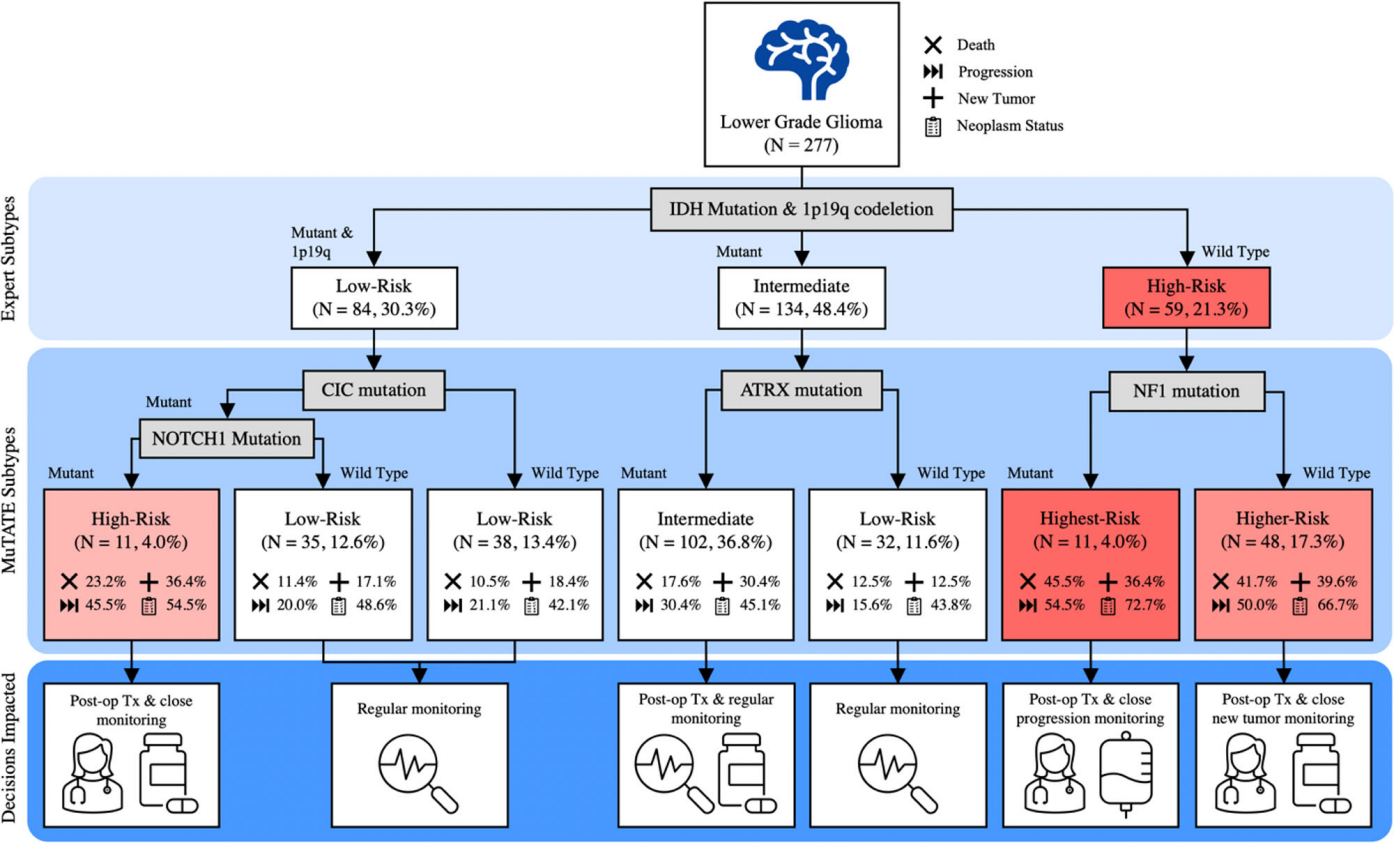
MuTATE identifies CIC and NOTCH1, ATRX, and NF1 as key markers of heightened LGG severity, enhancing risk classification and potentially reshaping clinical decision-making. The MuTATE-generated multi-endpoint decision tree stratifies LGG patients first by IDH1 and 1p19q status (top section, aligned with expert subtypes), and then by CIC, NOTCH1, NF1, and ATRX mutation status (middle section, representing MuTATE-defined final subtypes). To interpret this figure, follow each branching path from the expert subtype (top) to the MuTATE-defined subgroups (middle). Each node summarizes how the presence or absence of specific mutations alters risk across endpoints. Icons and color gradients reflect increasing clinical severity and inform potential clinical actions (bottom). Each node includes the number of patients (N, %) and subtype-specific percentages for key clinical endpoints: mortality, progression, new tumor events, and neoplasm status. Color shading reflects estimated clinical severity, with darker shades indicating higher-risk subtypes (defined by rates of death, progression, new tumor events, and neoplasm status). Summary statistics (% of patients with each clinical outcome) are provided for each subtype to illustrate clinical heterogeneity. Statistical associations between MuTATE subtypes and clinical outcomes were evaluated using logistic and Cox regression models and are reported in Fig. S4 and Supplementary Data 4–6. These results demonstrate MuTATE’s ability to replicate expert-defined classifications (e.g., IDH1-1p19q) while revealing more granular, higher-risk subgroups that were not captured by existing clinical models. The bottom section of the figure summarizes potential implications for clinical decision-making based on MuTATE-defined subtypes, including options for tailored monitoring, therapeutic escalation, or intensified surveillance. Notably, CIC and NOTCH1 variants stratified a higher-risk group within the traditionally low-risk IDH-mutant population, while NF1 and ATRX variants flagged more aggressive disease courses—highlighting MuTATE’s potential to inform post-resection therapeutic decisions and targeted surveillance strategies.

**Fig. 4:**
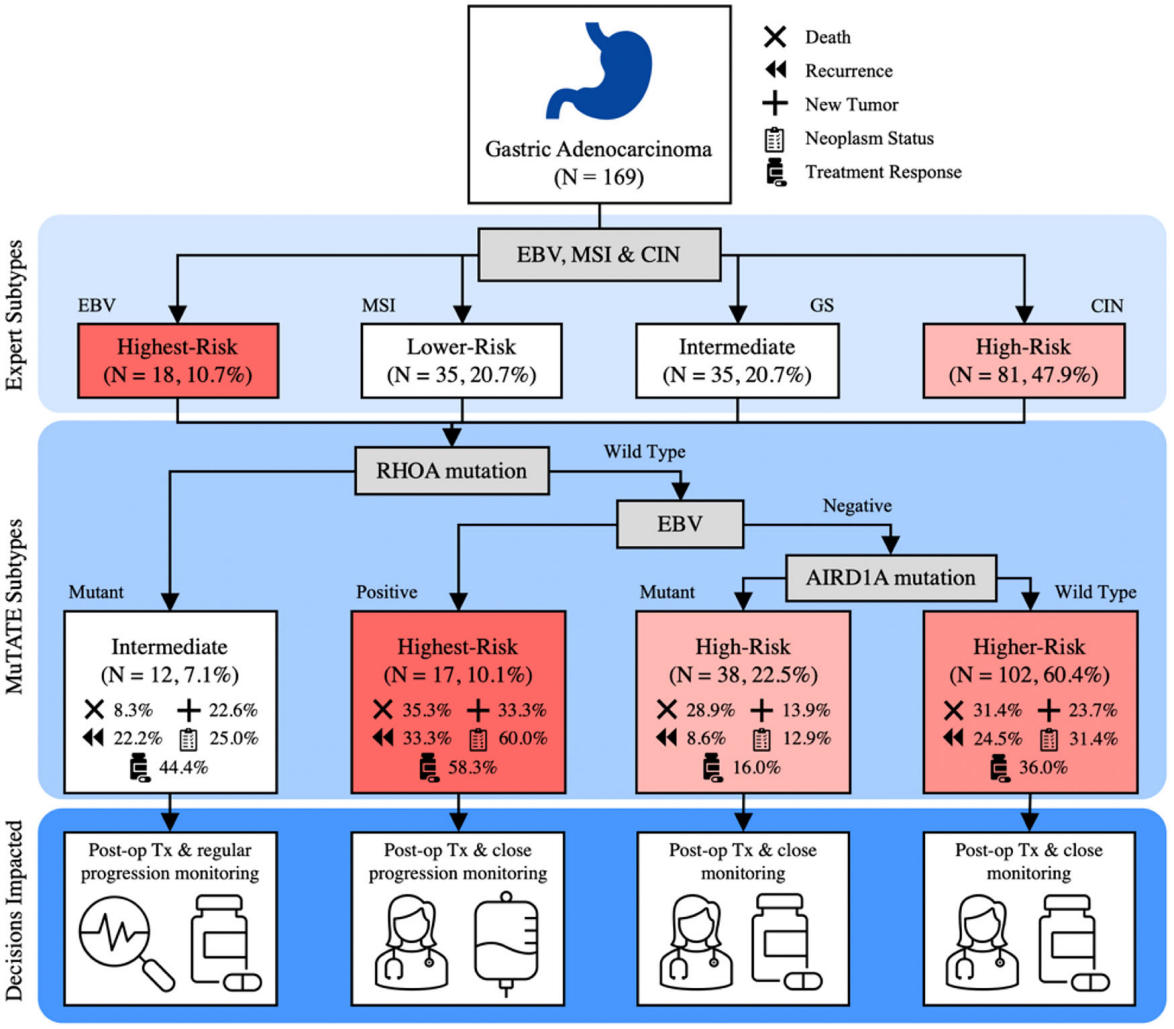
MuTATE identifies RHOA and ARID1A, as key markers of GA severity, enhancing classification of clinical phenotype and treatment response and potentially reshaping clinical decision-making. The top section illustrates the expert-defined GA subtypes based on EBV, MSI, CIN, and GS classifications, including corresponding patient counts and risk categories. The middle panel shows the MuTATE-derived multi-endpoint decision tree, which first stratifies patients by RHOA and EBV status, then further divides subgroups based on ARID1A mutation status—revealing clinically distinct phenotypes overlooked by expert-defined GA subtypes. To interpret this figure, follow each branching path from the expert subtype (top) to the MuTATE-defined subgroups (middle). Each node summarizes how the presence or absence of specific mutations alters risk across endpoints. Icons and color gradients reflect increasing clinical severity and inform potential clinical actions (bottom). Each node displays the number of patients (*N*, %), and subtype-specific percentages for key clinical endpoints: mortality, recurrence, new tumor events, neoplasm status, and treatment response. Color shading reflects clinical severity, with darker shades indicating higher-risk subtypes (defined by rates of death, recurrence, new tumor events, neoplasm status, and treatment response). MuTATE identified subgroups with markedly worse outcomes—such as RHOA-mutant GA with high rates of non-remission and recurrence, and ARID1A wild-type tumors within the traditionally “genomically stable” group—highlighting hidden risk missed by expert models. The bottom section outlines potential treatment implications, such as intensified post-operative therapy and closer surveillance. These findings underscore MuTATE’s ability to uncover granular, prognostically meaningful subtypes that could support more personalized and equitable treatment decisions in GA. Statistical associations were evaluated using logistic and Cox regression (Fig. S5, Supplementary Data 4–6). EBV Epstein Barr-Virus, MSI microsatellite instability, CIN chromosomal instability.

**Fig. 5:**
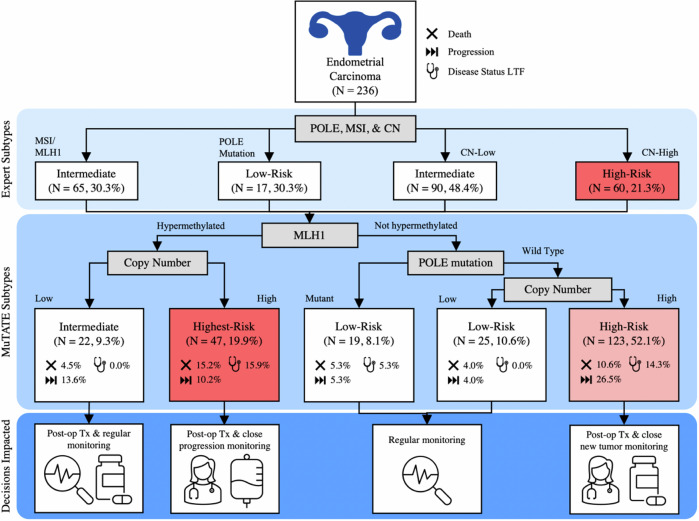
MuTATE identifies copy number and MLH1 hypermethylation as key markers of EC severity, enhancing classification of clinical phenotype and treatment response and potentially reshaping clinical decision-making. The top section presents expert-defined EC subtypes based on POLE, MSI, MLH1, and copy number (CN) classifications, including corresponding patient counts and risk categories. The middle panel shows the MuTATE-derived multi-endpoint decision tree, which first stratifies patients by MLH1 hypermethylation status, followed by copy number alterations (for hypermethylated patients), and POLE and CN status (for non-hypermethylated patients). To interpret this figure, follow each branching path from the expert subtype (top) to the MuTATE-defined subgroups (middle). Each node summarizes how the presence or absence of specific mutations alters risk across endpoints. Icons and color gradients reflect increasing clinical severity and inform potential clinical actions (bottom). Each node reports the number of patients (*N*, %) and subtype-specific percentages for key clinical endpoints: mortality, progression, and disease status. Color shading reflects estimated clinical severity, with darker red tones indicating higher-risk subtypes based on endpoint distributions. MuTATE uncovered high-risk subgroups that were not visible in expert-defined models. Notably, patients with MLH1 hypermethylation and high copy number alterations—traditionally considered “intermediate-risk”—exhibited markedly worse outcomes, including the highest observed rates of death, persistent disease, and progression. Similarly, patients with high CN and wild-type POLE status among the MLH1 non-hypermethylated group were reclassified as high-risk, despite not being flagged by expert models. These findings underscore MuTATE’s capacity to reveal granular, multi-endpoint phenotypes that may better inform post-operative risk stratification, treatment intensification, and surveillance strategies. The bottom panel summarizes potential clinical implications for each MuTATE-defined subtype, ranging from routine monitoring to close progression and new tumor surveillance. Statistical associations between MuTATE subtypes and clinical outcomes were evaluated using logistic and Cox regression and are reported in Fig. S6 and Supplementary Data 4–6. CN copy number, MSI microsatellite instability, LTF lost-to-follow-up.

MuTATE molecular models for LGG, GA, and EC have revealed novel molecular signatures when validated against manually-constructed established models, augmenting the existing clinical understanding of these diseases (Figs. 3–5, Figs. S4–6). Figure 3 provides a visual comparison of expert-derived vs. MuTATE-derived LGG subtypes, helping readers understand how MuTATE refines patient stratification. The middle panel illustrates how mutation status (e.g., CIC, NOTCH1) further stratifies risk within groups defined by traditional markers like IDH and 1p19q. The color gradient reflects increasing clinical severity across endpoints. In the LGG cohort, MuTATE identified *CIC* and *NOTCH1* (HGNC:7881), *ATRX*, and *NF1* (HGNC:7765) as key markers of heightened LGG severity, augmenting risk stratification and potentially reshaping clinical strategies, while also successfully recognizing the previously-established manual expert partitions on *IDH1* variant and 1p19q codeletion (Fig. 3, Fig. S4). Surprisingly, patients initially classified as “low-risk” in established manual models due to *IDH* variant and 1p19q codeletion LGG, may face a higher risk of severe disease (23.2% mortality, 45.5% progression, 36.4% new tumor) if they harbor concurrent *CIC* and *NOTCH1* variants (inactivating molecular alterations associated with 1p19q codeletion), signifying the need for intensified monitoring and personalized therapeutic strategies. *NF1* variant (an inactivating molecular alteration associated with *IDH1* wild-type) emerged as an indicator of aggressive disease (45.5% mortality, 54.5% progression, 36.4% new tumor) in patients with *IDH* wild-type LGG, underscoring the imperative for tailored therapeutic approaches and vigilant surveillance. Lastly, *ATRX* variant (an inactivating molecular alteration associated with *IDH* variant) correlates with increased disease progression (30.4%) in *IDH* variant LGG, reinforcing MuTATE’s role in refining risk paradigms and personalized treatment in LGG.

Figure 4 highlights MuTATE’s ability to uncover clinically relevant subtypes in GA that were overlooked by expert models. For example, MuTATE distinguishes a high-risk ARID1A wild-type subgroup within the genomically stable category, shown in darker red to reflect increased recurrence and disease progression. In the GA cohort, MuTATE expanded upon the expert model by identifying EBV, *RHOA*, and *ARID1A* as key indicators of GA severity, enriching clinical phenotype and treatment response classification and reshaping clinical decision-making (Fig. 4, Fig. S5). MuTATE identified EBV (the primary GA expert partition), *RHOA* (frequently mutated in the “genomically stable” expert subtype) and *ARID1A* (a GA tumor suppressor^46^), revealing novel clinical stratifications that were not captured by the expert model. Remarkably, individuals with *RHOA* variant GA (including patients from all expert subtypes) exhibited a strikingly high progression rate (44.4% non-remission) during treatment, revealing a critical clinical paradox of robust survival alongside elevated disease aggressiveness, thus highlighting a pressing need for tailored therapeutic interventions. This progression was under-recognized in the expert model, demonstrating MuTATE’s potential to highlight key clinical differences. EBV signaled severe disease (35.3% mortality, 58.3% non-remission), aligning with the EBV expert subtype, warranting targeted therapies and vigilant surveillance among those with RHOA wild-type and EBV-positive GA. Crucially, MuTATE uncovered novel insights regarding *ARID1A* status in GA. Individuals harboring *ARID1A* wild-type GA exhibited elevated rates of mortality (31.4%), recurrence (24.5%), new tumors (23.7%), and progression (36% non-remission) during treatment, suggesting that the *ARID1A* wild-type group, previously overlooked by the expert model, represents a higher-risk category that should be monitored for timely interventions. Notably, most patients in the GS expert subtype, were reclassified into the higher-risk *ARID1A* wild-type group, indicating that the expert model underestimated the clinical severity of these patients. This reclassification suggests that ARID1A wild-type status may define a previously overlooked high-risk subgroup in GA.

Figure 5 shows how MuTATE reclassifies EC patients by incorporating copy number and MLH1 methylation status. Patients previously considered intermediate-risk based on expert models are reclassified into a highest-risk group, reflected in the red-shaded node under the “MLH1 hypermethylated + high CN” branch. In the EC cohort, MuTATE identified copy number (CN), *POLE*, and *MLH1* hypermethylation as key markers of severity, enriching clinical phenotype classification and potentially reshaping clinical decision-making (Fig. 5, Fig. S6). Strikingly, among EC patients typically regarded as “intermediate-risk” in established manual models due to MLH1 hypermethylation, those with concurrent high CN exhibited not only the lowest survival (15.2% mortality) rates but also the highest incidence of persistent disease (15.9%) and a higher progression rate (10.2%), casting a spotlight on a previously underestimated high-risk EC subgroup for intensified monitoring and specialized treatment strategies. *POLE* variant signaled less severe disease in *MLH1* not hypermethylated EC, aligning with the *POLE* expert subtype and reinforcing the robustness of our classifications. High CN signals severe disease (10.6% mortality, 26.5% progression, 14.3% persistent disease) in those with *POLE* wild-type and *MLH1* not hypermethylated EC, aligning with the CN-high expert subtype, warranting tailored therapeutic approaches and vigilant surveillance. Lastly, low CN signals the least severe disease in *POLE* wild-type and *MLH1* not hypermethylated EC, suggesting less severity than the expert subtype and underscoring MuTATE’s ability to capture multiple endpoints for clinical decision-making.

### MuTATE subtypes are prognostically informative

MuTATE partitions and the biomarkers they identify are independent prognostic markers of disease, defining subtypes associated with multiple endpoints, revealing novel phenotype heterogeneity in all cohorts.

In LGG, the partition on *ATRX* variant predicted new tumor events (aOR=2.89, 95%CI: 1.00–10.54, *p* = 0.071) compared to those without *ATRX* variant among patients with *IDH1* variant (Fig. S4). In multivariable biomarker analyses, *IDH1* variant protected against death (aOR = 0.33, 95%CI: 0.14–0.76, *p* = 0.010; aHR = 0.39; 95%CI: 0.20–0.78, *p* = 0.008), progression (aOR=0.33, 95%CI: 0.14–0.74, *p* = 0.007; aHR = 0.36, 95%CI: 0.20–0.65, *p* = 0.001), and new tumor events (aHR=0.37, 95%CI: 0.20–0.71, *p* = 0.003) (Fig. S7b, Supplementary Data 6–7). 1p19q codeletion protected against death (aHR=0.19, 95%CI: 0.05–0.66, *p* = 0.009) and progression (aHR = 0.35, 95%CI: 0.15–0.85, *p* = 0.020), while *NF1* variant predicted death (aHR = 2.50, 95%CI: 1.08–5.79, *p* = 0.032). In multivariable LGG subtype analyses, the *IDH1* wild-type and *NF1* variant subtype predicted death (aOR = 7.08, 95%CI: 1.49–37.09, *p* = 0.015; aHR=15.08, 95%CI: 3.87–58.78, *p* < 0.001), progression (aOR = 10.12, 95%CI: 1.92–79.21, *p* = 0.011; aHR = 8.44, 95%CI: 2.82–25.23, *p* < 0.001), and new tumor events (aHR=6.69, 95%CI: 1.88–23.74, *p* = 0.003) compared to the 1p19q codeletion and *CIC* wild-type subtype (Fig. S7a, Supplementary Data 4–5). The *IDH1* wild-type and *NF1* wild-type subgroup showed significant lower risk of these outcomes and were more likely to remain “with tumor” (aOR = 3.00, 95%CI: 1.18–7.91, *p* = 0.023), suggesting *NF1* variant marks increased risk of severe disease in the already high-risk *IDH1* wild-type LGG subtype.

In GA, the partition on EBV+ predicted poor treatment response (aOR = 3.12, 95%CI: 0.92–11.27, *p* = 0.069) and remaining with tumor (aOR = 4.16, 95%CI: 1.39–13.33, *p* = 0.012) compared to those who were EBV- among patients with *RHOA* wild-type (Fig. S5). The EBV biomarker was also significantly associated with neoplasm status (aOR = 4.63, 95%CI: 1.51–15.22, *p* = 0.008) and poor response to treatment (aOR = 4.31, 95%CI: 1.17–17.62, *p* = 0.031) (Fig. S7b, Supplementary Data 6–7). *ARID1A* variant protected against recurrence (aOR=0.29, 95%CI: 0.07–0.91, *p* = 0.056), poor treatment response (aOR = 0.34, 95%CI: 0.09–1.00, *p* = 0.069), and remaining with tumor (aOR=0.32, 95%CI: 0.09–0.93, *p* = 0.053) compared to those with *ARID1A* wild-type among patients with *RHOA* wild-type and EBV-. Multivariable subtype analyses showed significantly increased odds of remaining with tumor in the *RHOA* wild-type and EBV+ subtype (aOR = 3.28, 95%CI: 1.08–10.67, *p* = 0.039) compared to the *RHOA* wild-type, EBV-, and *ARID1A* wild-type subtype (Fig. S7a, Supplementary Data 4–5). Results suggest *RHOA* wild-type, EBV- and *ARID1A* variant GA has better prognosis, while the *RHOA* wild-type EBV+ subtype has poorer prognosis.

In EC, the partition on high CN cluster predicted progression (aOR=8.65, 95%CI: 1.71–157.88, *p* = 0.038) compared to those with lower CN clusters among patients without *MLH1* hypermethylation and with *POLE* wild-type (Fig. S6). In multivariable biomarker analyses, high CN cluster was associated with increased risk of death (aOR = 4.02, 95%CI: 1.10–26.01, *p* = 0.070; aHR = 3.59, 95%CI: 0.83–15.51, *p* = 0.087) and predicted progression (aOR = 4.07, 95%CI: 1.52–14.19, *p* = 0.011; aHR = 2.97, 95%CI: 1.04–8.45, *p* = 0.042) (Fig. S7b, Supplementary Data 6–7). While *POLE* variant had a protective univariable association with progression (aOR = 0.27, 95%CI: 0.04–0.96, *p* = 0.085), this was not observed in multivariable analyses, suggesting CN cluster and *MLH1* hypermethylation status explain some of this association. Multivariable EC subtype analyses also showed significant associations across clinical endpoints (Fig. S7a, Supplementary Data 4–5). The *MLH1* non-hypermethylated, *POLE* wild-type, and low CN subtype protected against progression (aOR = 0.12, 95%CI: 0.01–0.58, p = 0.038; aHR = 0.17, 95%CI: 0.02–1.23, *p* = 0.079) compared to the *MLH1* non-hypermethylated, *POLE* wild-type, and CN high subtype, the most prevalent EC subtype. The *MLH1* non-hypermethylated and *POLE* variant subtype protected against progression (aOR = 0.15, 95%CI: 0.01–0.80, *p* = 0.075).

MuTATE models consistently exhibited superior performance compared to CART, resulting in significantly enhanced accuracy, and improved true and false discovery rates, as corroborated by cross-validation analyses of biopsies from three clinical cohorts. MuTATE surpassed CART, highlighting its substantial potential to advance our comprehension of clinical scenarios. Furthermore, the MuTATE molecular models developed for LGG, GA, and EC unveiled innovative molecular signatures, enriching the established clinical models. MuTATE’s partitioning approach and the biomarkers it identifies emerged as independent prognostic indicators of disease, characterizing subtypes associated with multiple clinical endpoints and unveiling previously unexplored phenotype heterogeneity across all cohorts.

Together, these results illustrate how MuTATE not only automates subtype discovery and risk classification, but also exposes molecularly defined subgroups missed by current expert paradigms. These insights may directly inform precision medicine strategies and guide post-operative management in LGG, GA, and EC.

## Discussion

We present MuTATE, an interpretable multi-endpoint decision tree algorithm that automates molecular subtyping and advances precision oncology by uncovering clinically actionable disease subtypes. By automating feature selection, ranking across multiple clinical endpoints, and statistically robust decision-making, MuTATE advances explainable modeling for precision oncology and uncovers novel molecular signatures. MuTATE consistently outperformed traditional ML models and expert-derived approaches across 18,400 simulations and validation in 682 TCGA patient biopsies, delivering superior accuracy and interpretability. Importantly, MuTATE’s interpretable outputs enable clinicians to visualize inclusion criteria and biomarker interactions, enabling equitable and scalable approach to risk stratification in precision oncology, particularly in settings where expert derived models are unavailable or biased.

Despite recent progress in integrating molecular biomarkers into cancer classification, key challenges persist—particularly in modeling complex diseases across multiple clinical endpoints. Existing approaches often suffer from overfitting, limited explainability, or reliance on manual feature curation, constraining their clinical utility and scalability. These limitations are exacerbated in diverse populations, where biased or labor-intensive models may not generalize. There is a clear need for automated, interpretable tools that model disease heterogeneity across endpoints while supporting actionable, equitable clinical decisions.

MuTATE addresses this need by providing a unified, interpretable model that supports heterogeneous data types, ranks features across multiple clinical endpoints, and integrates statistical significance testing at each split. Prior methods—including generalized linear mixed models (GLMMs), ensemble classifiers, deep learning, and hierarchical multi-label classifiers—have explored multi-endpoint prediction, but none combine multi-modal input, flexible feature selection, and visual interpretability in a clinically actionable framework^47–54^. While GLMM-based approaches, such as TMBcat and THOR, offer statistically rigorous multi-endpoint modeling with random effects and penalized fusion to handle subgroup heterogeneity, they rely on strong distributional assumptions, require extensive tuning, and are computationally intensive^47,48^. These models are designed for hypothesis-driven inference rather than real-time clinical use, and their outputs lack the intuitive interpretability necessary for direct integration into clinical workflows. In contrast, MuTATE flexibly discovers nonlinear interactions across mixed endpoint types and produces visually intuitive, human-readable subtypes that align with clinical reasoning. By avoiding black-box architectures and restrictive model forms, MuTATE supports scalable, explainable risk stratification and is well suited for practical deployment in multidisciplinary tumor boards and electronic health record systems. In future work, we plan to benchmark MuTATE against ensemble and kernel-based methods such as random forests and support vector machines, to quantify trade-offs in performance versus explainability. We note that direct benchmarking with random forests and support vector machines was not conducted in this study because these models lack unified, interpretable tree structures required for multi-endpoint risk stratification. Such comparisons are planned in future work to further evaluate trade-offs in performance and explainability. However, unlike these models, MuTATE produces a single, interpretable tree explicitly suited for multi-endpoint modeling. In real-world patient data, MuTATE successfully identified known biomarkers and revealed novel molecular signatures with prognostic relevance across LGG, EC, and GA. While MuTATE was validated on three TCGA cohorts with established expert-derived decision trees (LGG, GA, and EC), these represent the only such cohorts in the TCGA database. Future work will extend MuTATE to additional cancer types and non-TCGA datasets to assess broader generalizability. For instance, in LGG, MuTATE replicated the established IDH and 1p19q partitions and identified new risk-informing variants, including CIC, NOTCH1, NF1, and ATRX. In GA, MuTATE recognized EBV status as a primary stratifier, while uncovering clinically meaningful partitions based on RHOA and ARID1A status. In EC, MuTATE confirmed the role of MLH1 hypermethylation and POLE variants, while revealing CN as an additional driver of disease severity.

These findings underscore MuTATE’s potential to refine molecular subtyping in cancer and improve patient risk stratification. For example, patients with IDH variant and 1p19q codeletion LGG—typically labeled “low-risk”—were reclassified as higher-risk when CIC and NOTCH1 variants were present. NOTCH1, in particular, has been implicated in promoting glioma stemness and invasion, and treatment resistance, suggesting its presence may portend more aggressive disease even in otherwise favorable molecular backgrounds. Similarly, NF1 variant conferred elevated risk within the already high-risk IDH wild-type LGG group. NF1, a negative regulator of the RAS/MAPK pathway, has been associated with poorer survival and mesenchymal transition in gliomas, highlighting its role in driving progression in the absence of canonical favorable markers. In GA, patients with ARID1A wild-type tumors—largely falling within the genomically stable (GS) subtype—demonstrated worse clinical outcomes than suggested by expert models. This inversion of expected risk highlights how MuTATE can uncover hidden molecular drivers: while ARID1A loss has classically been associated with aggressive gastric phenotypes, our findings suggest that ARID1A wild-type status may also define a distinct high-risk subgroup, especially in the context of RHOA wild-type and EBV-positive disease. In EC, high CN status in MLH1-hypermethylated tumors signaled a severe subtype previously considered intermediate-risk. This finding is clinically actionable, as CN status is rarely considered when MLH1 methylation is used to stratify risk—MuTATE offers a more nuanced view that may better inform treatment intensity.

In clinical practice, such reclassifications could prompt earlier imaging surveillance or consideration for adjuvant therapy in patients currently deemed low-risk by traditional models. These reclassifications suggest that MuTATE can uncover hidden risk within presumed low- or intermediate-risk groups, refining subtype risk models with direct implications for treatment selection and surveillance planning. For instance, a patient with LGG harboring CIC and NOTCH1 mutations—despite traditionally favorable IDH-1p19q status—could be reclassified as higher-risk using MuTATE, prompting earlier imaging or consideration for adjuvant therapy. Importantly, these results were derived through an automated, interpretable framework that mitigates subjective expert bias and enables scalable clinical integration.

The clinical relevance of MuTATE-identified biomarkers is further reinforced by their alignment with prior literature across tumor types. Studies found *NOTCH1* variants were associated with shorter survival in glioma^55^, and the Notch1 signaling pathway was found to promote self-renewal and invasion in glioma initiating cells^56^. While our findings align with early work showing that *ATRX* loss was associated with longer survival in astrocytic tumors^57^, our results also indicated associations with increased disease progression and new tumors, highlighting how ATRX status may have differential impact across clinical endpoints. *MAPK* pathway gene alterations (frequently affecting NF1) and *ATRX* variants or expression loss were also common molecular alterations in *IDH* wild-type anaplastic astrocytoma with piloid features^58,59^. *RHOA* variants were found to promote metastasis in clinical and mouse models of GA^60–62^, and *PIK3CA*, *ARID1A* and *RHOA* variants were enriched in inflamed GA^63^, supporting earlier work showing TP53 and ARID1A variants were less common in intestinal metaplasia than in advanced gastric cancer^64^. Recent research also found EBV contributes to GA oncogenesis^65^, supporting earlier work^7^. In EC, the *MLH1* methylation phenotype was associated with lower disease-specific survival^66^, while *POLE* variants define the ultramutated EC subtype with favorable prognosis^67^. CN alterations are emerging as critical EC biomarkers, with therapeutic implications for genomic instability^68^.

MuTATE identified these associations without prior assumptions or manual curation, demonstrating its capability to independently recapitulate and refine clinically actionable subtypes across molecular and phenotypic dimensions. In doing so, MuTATE offers a framework that may bridge the gap between algorithmic precision and clinical interpretation, offering a pathway toward scalable, explainable, and equitable decision support for complex diseases.

We rigorously benchmarked MuTATE against CART across 18,400 simulations, systematically varying the number of targets, features, sample sizes, and inter-target correlations. MuTATE consistently outperformed CART, demonstrating higher predictive accuracy, lower false discovery rates, and superior model interpretability. Performance improved with increasing sample size and model depth, while CART exhibited signs of overfitting. Notably, MuTATE maintained robustness in settings with strong target correlation, showcasing its ability to model interdependent outcomes. To ensure methodological rigor and generalizability, MuTATE was evaluated using repeated cross-validation, held-out test sets, and multivariable regression analyses across both simulations and clinical cohorts. These statistical procedures provide robust evidence for MuTATE’s reliability, predictive validity, and real-world applicability. Compared to GLMMs and other regression-based approaches—which require prespecified variable–outcome relationships and are limited to fixed endpoint types—MuTATE automatically discovers nonlinear interactions across mixed outcome types within a unified, statistically robust framework. While GLMMs allow statistical interpretability, they are constrained to pre-specified relationships and lack the flexible, data-driven structure MuTATE offers for multi-modal risk modeling. This flexibility and explainability make MuTATE well-suited for biomarker discovery in diverse clinical contexts.

Unlike ensemble or deep learning methods that often require separate models for each outcome, MuTATE generates a single, interpretable decision tree that integrates multiple clinical endpoints. This unified structure enables clinicians to visualize inclusion criteria and biomarker interactions in a format aligned with clinical reasoning. By avoiding black-box architectures and rigid linear assumptions, MuTATE balances flexibility and transparency—critical for real-world implementation in clinical decision-making workflows. In contrast to GLMMs, which require separate, prespecified models per endpoint and assume linear relationships, MuTATE offers unified, nonparametric modeling across mixed outcome types. Tree ensembles such as random forests may improve prediction but lack cohesive, interpretable subtypes, limiting their clinical actionability. Hierarchical multi-label classifiers support multi-endpoint prediction but often forgo statistical rigor at decision splits. MuTATE fills this methodological gap by offering a single, statistically validated, interpretable model purpose-built for clinical integration.

While MuTATE outperformed CART in synthetic benchmarks, some limitations warrant consideration. Performance degradation with increasing feature count may reflect synthetic data artifacts, such as spurious associations. Simulations did not model real-world noise, such as variant misclassification or missing clinical data. Although we opted not to impute missing values to preserve clinical fidelity, real-world deployment will require strategies to handle incomplete data, particularly in resource-limited settings. While multiple imputation is a valid alternative, we opted for a simpler exclusion approach given the dataset size, low proportion of missingness, and our priority to maintain model interpretability and avoid introducing imputation bias. As with many TCGA-based studies, batch effects and population biases may limit generalizability. We acknowledge known biases in TCGA, including overrepresentation of White and European-ancestry populations, and absence of batch correction across institutions. These factors may limit generalizability in underrepresented groups. Future work will validate MuTATE in more diverse, prospective cohorts. While our internal validation using cross-validation across three TCGA cohorts demonstrates model robustness, external validation using independent, multicenter datasets remains an important future direction to further confirm generalizability. While TCGA-LGG, GA, and EC are the only cohorts with published expert trees to support validation, future work will apply MuTATE to non-TCGA, multicenter, and prospective datasets to assess generalizability across diverse populations and care settings. We also plan to evaluate MuTATE in routine clinical workflows and real-time decision support systems. MuTATE’s computational complexity scales linearly with the number of samples, features, and endpoints, allowing practical application in large clinical datasets. Model training and validation across our three cohorts were completed within a clinically feasible timeframe (e.g., under 24 h on standard hardware). Additionally, due to the lack of publicly available multi-target algorithms with interpretable tree structures, benchmarking was limited. While we focused on comparing MuTATE to CART due to its clinical interpretability, future work will incorporate comparisons with ensemble methods such as random forests or support vector machines to further assess performance trade-offs between accuracy and explainability. Nonetheless, our use of cross-validation and multivariable regression supports MuTATE’s reliability and generalizability.

MuTATE’s interpretable outputs make it suitable for real-world clinical integration, particularly in scenarios requiring transparent decision support for stratifying patients by prognosis or treatment eligibility. For example, an oncologist reviewing a patient with LGG could use a MuTATE-generated decision tree to understand how co-occurring mutations such as CIC and NOTCH1 influence risk despite otherwise favorable markers like IDH mutation and 1p19q codeletion. This transparency supports shared decision-making, enabling clinicians to explain how molecular features influence risk and treatment recommendations. Additionally, MuTATE’s model structure can be readily incorporated into multidisciplinary tumor boards, providing a unified framework for integrating diverse clinical endpoints into patient stratification workflows.

MuTATE can be integrated into clinical workflows to support real-time decision-making. Because MuTATE’s models are interpretable and directly visualizable, they are suitable for use in multidisciplinary tumor boards and could be incorporated into electronic health records to support real-time clinical decision-making. For example, the algorithm could be deployed in molecular tumor boards or embedded in pathology reporting systems to stratify patients across survival, recurrence, and treatment response. Its interpretable outputs can guide decisions around adjuvant therapy or imaging frequency, especially for ambiguous intermediate-risk cases. Future work will focus on prospective clinical validation, incorporation of multi-omic and treatment data, and deployment in settings lacking expert molecular resources. Together, MuTATE’s predictive accuracy and ability to uncover clinically relevant subtypes across LGG, GA, and EC underscore its value for real-world precision oncology. By automating expert-level insights and supporting multi-endpoint risk modeling, MuTATE bridges algorithmic rigor and clinical relevance. Future validation in diverse cohorts will be critical to realizing its translational potential.

In summary, MuTATE supports clinical decision-making by translating multi-endpoint molecular data into simple, interpretable rules. Its application enables clinicians to identify hidden high-risk subgroups, tailor treatment plans, and inform surveillance strategies based on molecular subtypes. This positions MuTATE as a scalable, equitable, and real-time decision support tool across oncology settings.

## Methods

### Tackling multi-endpoint explainability

Disease subtyping for precision medicine remains limited by the inability of most ML algorithms to model multiple clinical endpoints in a transparent, interpretable, and statistically rigorous manner. While black-box models such as deep neural networks may achieve strong predictive accuracy but lack transparency required for clinical deployment, and manual expert-derived models, though interpretable, are labor-intensive, subjectivity, and difficult to scalability. Moreover, existing ML approaches struggle to integrate multiple endpoint types (e.g., survival, binary, continuous) in a unified model, limiting their generalizability and clinical relevance.

To address these gaps, we benchmark MuTATE against commonly used modeling frameworks (Table 1). While GLMMs offer interpretability, they require separate models for each endpoint and assume linear relationships^47,48^. CART produces decision rules but lacks support for multi-endpoint modeling and is prone to overfitting^33^. Random forests and boosting algorithms scale to high-dimensional data but fail to provide clinically interpretable subtypes^50,51^. Hierarchical multi-label classification (HMC) supports multiple outputs but typically only handles categorical variables and lacks statistically grounded subgroup criteria^54^. In contrast, MuTATE uniquely combines rule-based interpretability with statistical control and cross-endpoint flexibility, offering a unified solution to multi-endpoint molecular modeling. MuTATE uniquely supports multi-endpoint statistical splitting, cross-endpoint feature ranking, and interpretable subtype inclusion criteria—capabilities lacking in GLMMs, tree ensembles, and deep learning methods.

**Table 1:** Qualitative comparison of MuTATE and benchmark modeling approaches across key features relevant to multi-endpoint disease subtyping

Feature/Model characteristic	MuTATE	CART	GLMM	Random Forests	Deep Learning	Hierarchical Multi-label Classification (HMC)
Handles multiple endpoints of different variable types	Yes	Limited	Limited	No	No	Partial
Interpretable decision rules	Yes	Yes	Partial	Limited	No	Limited
Supports heterogeneous data (e.g., categorical, continuous, molecular)	Yes	Partial	Partial	Yes	Yes	Limited
Guards against overfitting	Yes	Partial	Yes	Yes	Partial	Limited
Scalable to high-dimensional data	Yes	Partial	Limited	Yes	Yes	Limited
Cross-endpoint feature ranking	Yes	No	No	No	No	Partial
Statistical significance-aware splits	Yes	No	Yes	No	No	Limited
Clinically interpretable subtype definitions and inclusion criteria (whole model)	Yes	Yes	Partial	Limited	No	Limited
Unified multi-endpoint model (single tree)	Yes	No	No	No	No	No

See Supplementary Data 9 for additional details.

MuTATE is a novel decision tree algorithm purpose-built for high-dimensional molecular and clinical data with heterogeneous outcome types (e.g., binary, continuous, survival)^45^. It extends the classic decision tree framework^33^ to generate explainable multi-endpoint models that identify clinically meaningful biomarkers and patient subgroups across complex diseases. By automating (i) feature evaluation, (ii) multi-endpoint ranking, and (iii) partitioning (Fig. 1)^45^, MuTATE enables explainable and data-driven molecular subtyping. At each node, MuTATE calculates endpoint-specific information gain (IG) and incorporates *p*-value–based metrics to prioritize statistically meaningful splits. Flexible partitioning strategies allow adaptation to diverse clinical endpoints. This design balances statistical rigor and interpretability, reducing overfitting and greed-driven tree expansion while producing clinically actionable subtypes defined by clear decision rules. MuTATE explicitly incorporates statistical significance in feature selection through endpoint-specific *p*-value thresholds, and its performance is validated using hypothesis testing and cross-validation in both simulated and real-world datasets.

MuTATE selects partitions using novel, multi-endpoint aware criteria: average IG across targets (**avgIG**), highest IG in any target (**maxIG**), meaningful IG ($\ge \,$*IG*_*cutoff*_) in the most targets (**mostIG**), lowest average *p*-value of statistically significant ($\le \alpha$) IG (**avgPVal**), lowest p-value, weighted by number of targets with significant IG (**minPVal**), significant IG in the most targets (**mostPVal**), or a subtree lookahead examining projected multi-target error after the split (**splitError**) (Figs. S2–3)^45^. These partitioning strategies enable flexible parameter tuning across clinical endpoints while constructing a unified, clinically interpretable multi-target decision tree. The decision rules generated by MuTATE span multiple endpoints and define either partitioning splits or terminal leaf nodes. Partitioning stops when further splits fail to improve performance or the node size falls below a threshold. We opted for True/False Positive/Negative node assessments over tree-specific metrics like tree edit distance and the Adjusted Rand Index, as MuTATE’s smaller tree size allows for easier interpretation, and its novel nodes offer valuable biological and clinical insights beyond traditional models. MuTATE reduces model greed (i.e., overly aggressive splitting) and overfitting by considering multiple targets and target weights in objective, quantitative feature selection and model construction, enhancing discovery of subtypes and biomarkers important across dimensions of disease. MuTATE’s performance was assessed via repeated cross-validation and statistical hypothesis testing across both synthetic and real-world datasets.

MuTATE’s computational complexity scales linearly with the number of samples, features, and endpoints, enabling efficient application to high-dimensional clinical datasets. As the algorithm partitions data based on multi-endpoint information, the tree depth can increase depending on data heterogeneity. The runtime scales linearly with the number of samples and features, similar to other machine learning models like CART. However, because MuTATE evaluates multiple endpoints simultaneously, the runtime also scales linearly with the number of endpoints. MuTATE’s capacity to identify clinically relevant subgroups across multiple endpoints makes it well-suited for applications in biomarker discovery, patient stratification, and personalized therapeutic development. By uniting interpretability, cross-endpoint relevance, and flexible decision-tree modeling, MuTATE fills a key methodological gap in multi-endpoint clinical modeling that existing subtyping or statistical approaches have not fully addressed.

### Datasets

MuTATE has been applied to: new synthetic datasets created for this work and three molecular clinical datasets for LGG, GA, and EC. These datasets are described below. All clinical data used in this study are publicly available from The Cancer Genome Atlas (TCGA) via the NCI Genomic Data Portal.

### New multi-endpoint synthetic datasets

The evaluation of multi-endpoint explainability in ML methods for molecular modeling requires a dataset: (1) where discriminative features are known and (2) that replicates the complexity of multi-objective tasks with known dependencies across endpoints. Synthetic data whose structure is described by a multi-target tree were used to facilitate the development, testing and validation of MuTATE (Fig. 1). These synthetic datasets allowed us to experimentally assess decision tree goodness-of-fit and gain insights into model complexity, training dataset size, model error, and true and false discoveries^69^.

We used a positive definite covariance matrix and defined the correlation structure (*c*) to generate *t* synthetic multivariate normally distributed targets for sample size *n* (Fig. 1b). Synthetic features were generated separately, with 50% continuous, 30% binary, and 20% categorical. We sampled features with replacement, divided targets into leaf quantiles, and randomly assigned them to unique leaf nodes to define the ground truth (GT), resulting in multi-target tree-structured data with a known structure. Synthetic data were used for development, testing and validation of MuTATE (Fig. 1). Generated data included 100 to 1000 observations, 2 to 5 target variables, an inter-target correlation of 0 to 1, 10 to 100 features, and GT model depth of 0 to 5 (Supplementary Data 1).

By employing synthetic datasets, we ensured a controlled environment for evaluating MuTATE’s performance and its ability to handle multi-endpoint explainability. This synthetic data offered a versatile platform to examine different scenarios, explore model behavior, and assess interpretability. The synthetic datasets’ tunability allowed us to investigate the algorithm’s performance across various dimensions for testing and validation.

### Clinical cohorts and expert trees

After benchmarking MuTATE on synthetic datasets, we applied it to three independent TCGA clinical cohorts to evaluate real-world applicability and expert model replication. Curated clinical and molecular data from TCGA for LGG, GA, and EC were obtained via the NCI Genomic Data Portal^70^ and used to evaluate MuTATE’s performance against expert clinical models (Fig. 1b). Of the 33 cancers included in TCGA, only three have established clinical molecular decision tree models for validation with MuTATE, highlighting the need for validated methods that can build such clinical models for the remaining TCGA cancers and for other diverse cohorts around the world.

The LGG cohort comprised 277 biopsies with 59 molecular features (including *IDH* [HGNC:5382–5383], *TP53* [HGNC:11998], *ATRX* [HGNC:886], and *CIC* [HGNC:14214]) and 7 clinical endpoints (vital status, overall survival, new tumor, tumor-free survival, progression, progression-free survival, and neoplasm status) from adults with previously untreated LGG (WHO grades II and III) (Fig. S3a, Supplementary Data 2)^5^. The GA cohort included 169 biopsies with 19 molecular features (including *TP53*, *ARID1A* [HGNC:11110], *PIK3CA* [HGNC:8975], *KRAS* [HGNC:6407], and *RHOA* [HGNC:667]) and 8 clinical endpoints (vital status, overall survival, new tumor, tumor-free survival, recurrence, recurrence-free survival, neoplasm status, and treatment response) from patients with GA primary tumor tissue who had not received prior chemotherapy or radiotherapy (Fig. S3b, Supplementary Data 2)^7^. The EC cohort included 236 biopsies with 117 molecular features (including *POLE* [HGNC:9177], *PTEN* [HGNC:9588], *PIK3CA*, *ARID1A*, and *PIK3R1* [HGNC:8979]) and 5 clinical endpoints (vital status, overall survival, progression, progression-free survival, and neoplasm status) from patients with endometrioid and serous or mixed histology tumors (Fig. S3c, Supplementary Data 2)^6^.

The multi-omics LGG^5^ expert tree included *IDH* and 1p19q in patients who were previously untreated (Fig. 3, Fig. S4). Those with *IDH* variant and 1p19q codeletion displayed minimal progression to glioblastoma (GBM), while those without *IDH* variant had severe clinical outcomes (GBM). The multi-omics GA^7^ expert tree identified Epstein–Barr virus (EBV), microsatellite instability (MSI), chromosomal instability (CIN), and GS in patients with GA primary tumor tissue who had not received prior chemotherapy or radiotherapy; GS tumors were enriched for *RHOA* variants (Fig. 4, Fig. S5). While subtypes exhibited distinct clinical and molecular characteristics, they did not demonstrate survival differences. The multi-omics EC^6^ expert tree included *POLE*, MSI/*MLH1* (HGNC:7127) hypermethylation, and variant frequency in patients with endometrioid and serous or mixed histology tumors (Fig. 5, Fig. S6). Copy-number (CN) high tumors had worse progression-free survival and shared genomic features with ovarian serous and basal-like breast carcinomas.

### Statistical methods

All analyses were performed in R V.4.2. Synthetic data characteristics were summarized (Supplementary Data 1) and clinical cohorts were assessed for significant differences using ANOVA and two sample t-tests, and Chi-squared and Fisher’s exact tests (Supplementary Data 2). MuTATE is freely available at GitHub (https://github.com/SarahAyton/MuTATE) under the GPLv3 license.

#### Model performance and explainability

The strength of established clinical models lies in their interpretability and visual hierarchical structure. ML that seeks to generate similar models of disease must preserve interpretability; the decision tree method is best suited to generate an interpretable hierarchical decision model. While ensemble approaches have gained popularity due to their improved precision, they do so at the expense of model interpretability, as they base predictions off of many models and in a “black box” and do not produce a synthesized clinically interpretable model. While state-of-the-art ensemble methods can identify important predictive features for endpoint-specific models, their interpretability diminishes significantly when attempting to analyze multiple sets of important features across multiple endpoint-specific models simultaneously. The lack of a unified explainable model framework complicates the ability to draw meaningful and clinically actionable comparisons between methods.

In each simulation, 100 synthetic multi-target GT trees were constructed, synthetic sets were divided into train and test sets (60/40 data split), and grid search assessed MuTATE trees and CART (RPART Version 4.1.16)^71^ models (Fig. 1c). Each simulation altered one parameter while others remained constant (default parameters: *n* = 100, *x* = 10 features, *t* = 4 targets, depth *d* = 4, *c* = 0 inter-target correlation). Models were evaluated for test error (node-weighted average of normalized target loss), true discovery rate (TDR—proportion of splits in the GT also present in the constructed tree), and false discovery rate (FDR—proportion of splits not present in the GT that are present in the constructed tree) across model parameters. Multivariable regression assessed the comparative test error, TDR, and FDR performance of MuTATE vs. CART, adjusting for synthetic data parameters (Fig. 2, Supplementary Data 1).

To compare MuTATE and CART, we fit multivariable linear and logistic regression models with model performance metrics (e.g., test error, TDR, FDR) as the dependent variables. Independent variables included model type (MuTATE vs. CART) and simulation parameters (e.g., sample size, number of targets, number of features, inter-target correlation, model depth, and simulation run ID). Coefficients represent the average difference in performance metrics associated with each model type, adjusting for simulated data characteristics. *P*-values reflect two-sided hypothesis tests with significance defined at *α* = 0.05. All statistical comparisons were adjusted for simulation parameters, including sample size, number of targets, features, inter-target correlation, and model depth. Performance differences between MuTATE and CART were evaluated using multivariable linear and logistic regression, with 95% confidence intervals derived from model-based standard errors. All results were verified using repeated cross-validation and held-out test sets to confirm robustness.

#### Molecular signature automation analyses

Clinical cohorts with established manual expert models were used to assess MuTATE’s ability to automate expert architectures and enhance molecular insights. Variables missing data in over 50% of patients and any variables for which all patients had the same observed value were excluded. Patients missing data for at least 50% of variables were excluded. Multiple imputation was not performed in the preparation of these to avoid biasing real world clinical data for model development. To assess comprehensive expert tree replication, we included several clinical endpoints: overall survival, tumor-free survival, progression-free survival, recurrence-free survival, vital status, neoplasm status, new tumor event, recurrence, disease progression, and treatment response (Fig. S3, Supplementary Data 1). For each clinical cohort, we performed 10-fold cross-validation on the training set to tune MuTATE’s partitioning parameters. Parameter combinations were evaluated based on average test error across folds, and the best-performing combination was selected to train the final model on the full training data.

This tuning process involved a grid search across MuTATE’s multi-endpoint splitting criteria, including avgIG, maxIG, mostIG, avgPVal, minPVal, mostPVal, and splitError (Figs. S2, 3). During tuning, we balanced the trade-off between model accuracy (measured by test error and true discovery rate) and interpretability (measured qualitatively by tree depth, simplicity of decision rules, and redundancy of features). Parameter sets resulting in minimal test error and fewer, more intuitive tree splits were prioritized to ensure clinical usability. This multi-objective optimization strategy ensured that resulting models were both statistically robust and interpretable. Cross-validation was stratified to maintain target class distributions, and tuning focused on selecting parameters that preserved generalizability across diverse patient subgroups. This multi-objective optimization approach simultaneously prioritized accuracy across multiple clinical endpoints and interpretability. For each cohort, MuTATE balanced trade-offs by selecting partitioning strategies that minimized test error while maintaining tree simplicity, and ensured endpoint-specific relevance through p-value–based criteria. Final models reflected both statistical rigor and clinical usability, optimized for each dataset’s unique endpoint structure.

Performance metrics (test error, TDR, and FDR) were then assessed on the held-out test set. This procedure was repeated across clinical endpoints. Stratified folds preserved class distributions to prevent target imbalance during training. All models were assessed for testing error, TDR, and FDR, and average estimates and 95% CIs were estimated across tuned folds (Supplementary Data 3). Expert partitions were used as the GT for TDR and FDR calculations to assess expert tree replication. Tuned parameters defined trained models, which were applied to the full cohorts.

Final trees were assessed for prognostic significance of partitions, biomarkers, and subtypes. ANOVA, two sample t-tests (with equal-variances assumption), Chi-squared, and Fisher’s exact tests assessed subtype differences (Supplementary Data 4). Partitions were assessed in logistic and Cox proportional hazards models across clinical endpoints to quantify clinical differences between sibling nodes (nodes generated from a common parent node) (Figs. 3–5, Supplementary Data 5). Identified subtypes were evaluated for prognostic significance using logistic and Cox models, enabling interpretation of MuTATE’s clinical relevance. Logistic and Cox proportional hazard models assessed endpoint associations between identified subtypes and explored endpoint associations in identified biomarkers (Fig. S7, Supplementary Data 6–9).

To assess model goodness-of-fit and reliability, we evaluated test set performance for each trained model, reporting mean test error and its variance across datasets. For clinical datasets, we additionally examined node-level statistical associations (e.g., logistic regression and Cox proportional hazards models) between identified subtypes and clinical outcomes. Statistically significant associations between sibling nodes across endpoints provided external validation of partition relevance, supporting the goodness-of-fit of MuTATE’s learned structures. Together, these hypothesis tests, regression models, and cross-validation procedures ensure rigorous performance evaluation and support the reliability, statistical validity, and generalizability of MuTATE across both simulated and real-world clinical settings.

## Supplementary information

MuTATE Supplement

MuTATE Supplement Data

## Data Availability

The original clinical cohort data were obtained from NCI Genomic Data Commons. The aggregated data and analytic results presented in the results and figures are provided in the Supplemental Tables. The source code for MuTATE and instructions on how to use it are freely available at: https://github.com/SarahAyton/MuTATE.git.
