# Supplementary material for: MuTATE: an interpretable multi-endpoint machine learning framework for automated molecular subtyping in cancer
Source: Npj Health Syst. 2025 Jul 3;2:23. doi: 10.1038/s44401-025-00025-4 (PMC13354218; doi:10.1038/s44401-025-00025-4)
Supplement: Supplementary file 1 — MuTATE Supplement [file 44401_2025_25_MOESM1_ESM.pdf]

Exploring Cancer Risk: Evaluating a Machine Learning Framework for Multi-Endpoint Modeling to Advance Precision Medicine and Clinical Decision Support

Supplementary Material

Supplementary Data

Supplementary Data 1. Association between method and model performance in 18,400 synthetic multi-target dataset simulations.

Supplementary Data 2. Demographic and clinical characteristics of three clinical cohorts.

Supplementary Data 3. Average performance of each method in multi-target development datasets for three cancers performed using 10-fold cross-validation.

Supplementary Data 4. Clinical outcome characteristics for three cancer datasets stratified by decision tree-identified molecular subtype.

Supplementary Data 5. Univariable associations between multi-target decision-tree identified molecular partitions (compared with their sibling node) and clinical outcomes in three cancer datasets.

Supplementary Data 6. Multivariable associations between multi-target decision-tree identified molecular subtypes (compared with the most prevalent leaf node) and clinical outcomes in three cancer datasets.

Supplementary Data 7. Univariable associations between decision-tree identified molecular biomarkers and clinical outcomes in three cancer datasets.

Supplementary Data 8. Multivariable associations between decision-tree identified molecular biomarkers and clinical outcomes in three cancer datasets.

Supplementary Data 9. Extended comparison of MuTATE and benchmark models across key features relevant to multi-endpoint disease subtyping.

Additional Results

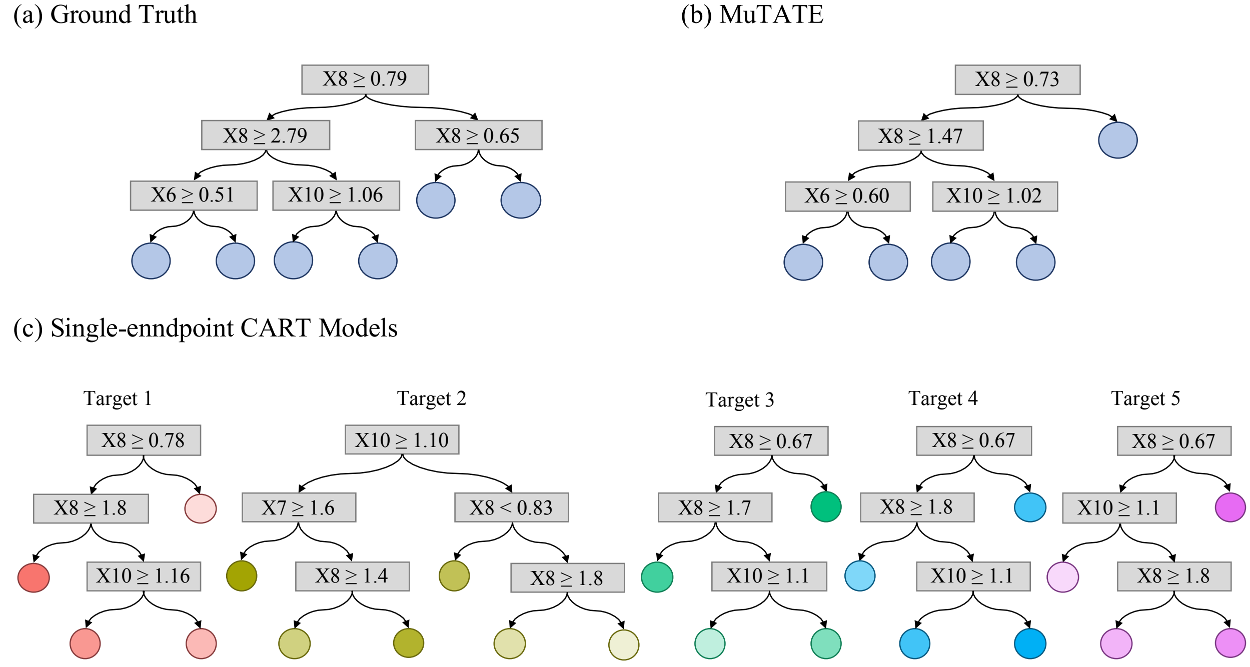

Supplementary Figure 1. An example of a ground truth tree (N = 100, depth = 3, no. targets = 5, c = 0, no. features = 10) used to generate simulated data (a), the multi-target model built on the training subset of simulated data (b), and the single-target CART models built on the training subset of simulated data (c).

(a)

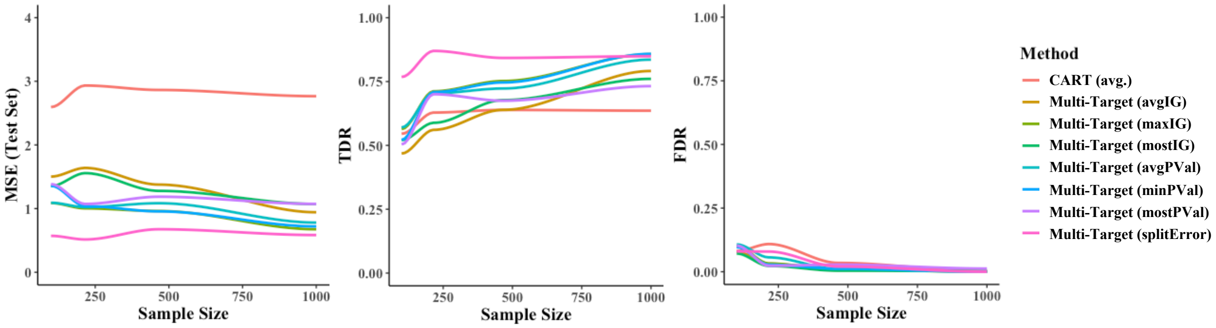
MSE (Test Set)
Sample Size
TDR
FDR
Method
CART (avg.)
Multi-Target (avgIG)
Multi-Target (maxIG)
Multi-Target (mostIG)
Multi-Target (avgPVal)
Multi-Target (minPVal)
Multi-Target (mostPVal)
Multi-Target (splitError)

(b)

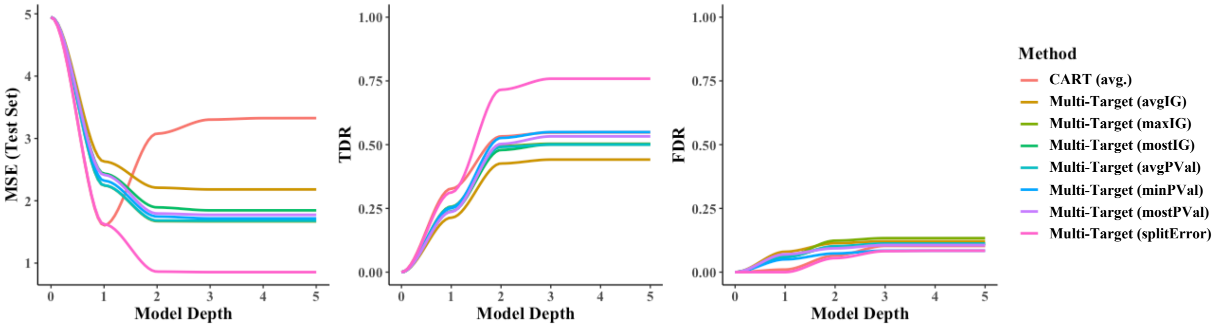
MSE (Test Set)
Model Depth
TDR
FDR
Method
CART (avg.)
Multi-Target (avgIG)
Multi-Target (maxIG)
Multi-Target (mostIG)
Multi-Target (avgPVal)
Multi-Target (minPVal)
Multi-Target (mostPVal)
Multi-Target (splitError)

(c)

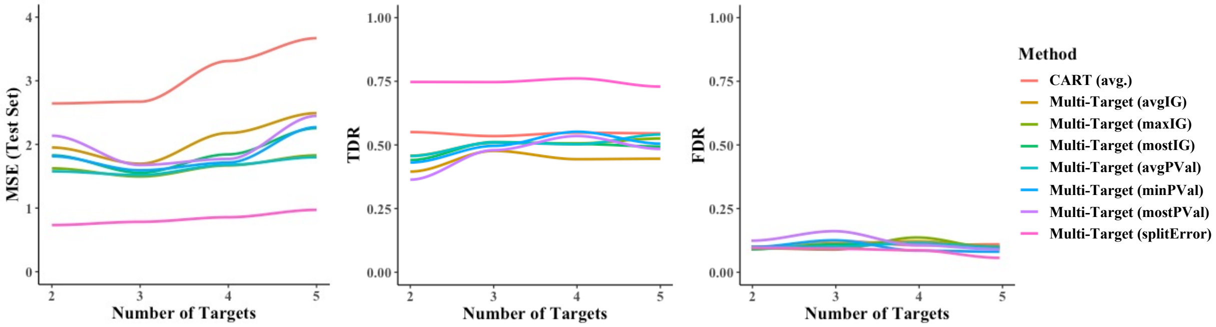
MSE (Test Set)
Number of Targets
TDR
FDR
Method
CART (avg.)
Multi-Target (avgIG)
Multi-Target (maxIG)
Multi-Target (mostIG)
Multi-Target (avgPVal)
Multi-Target (minPVal)
Multi-Target (mostPVal)
Multi-Target (splitError)

(d)

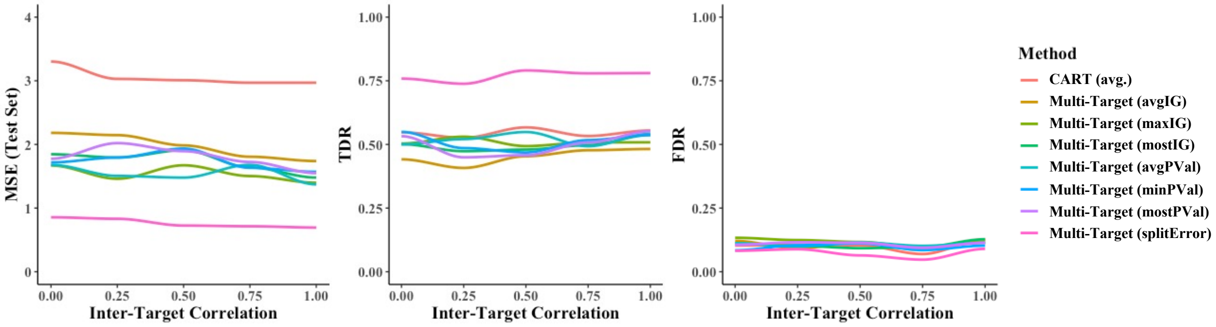
MSE (Test Set)
Inter-Target Correlation
TDR
FDR
Method
CART (avg.)
Multi-Target (avgIG)
Multi-Target (maxIG)
Multi-Target (mostIG)
Multi-Target (avgPVal)
Multi-Target (minPVal)
Multi-Target (mostPVal)
Multi-Target (splitError)

(e)

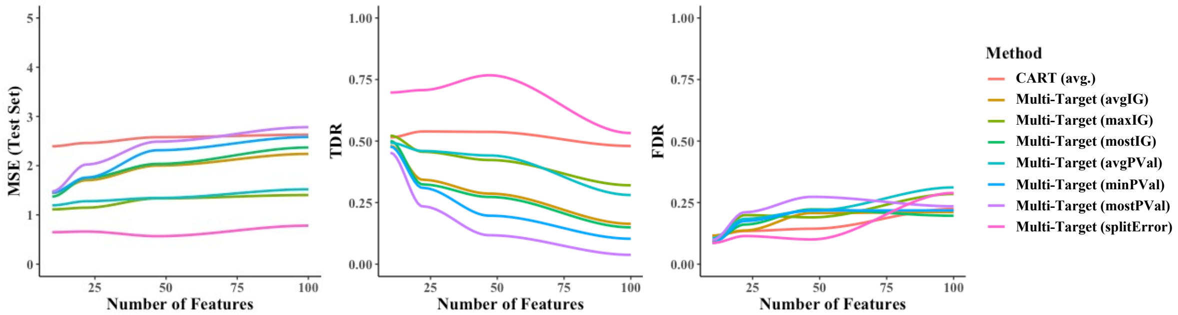

Supplementary Figure 2. Simulation experiments showing the influence of sample size (a), model depth (b), number of targets (c), inter-target correlation (d), and number of features (e) on multi-target decision tree performance using the average of single-target CART models and using MuTATE under different splitting criteria.

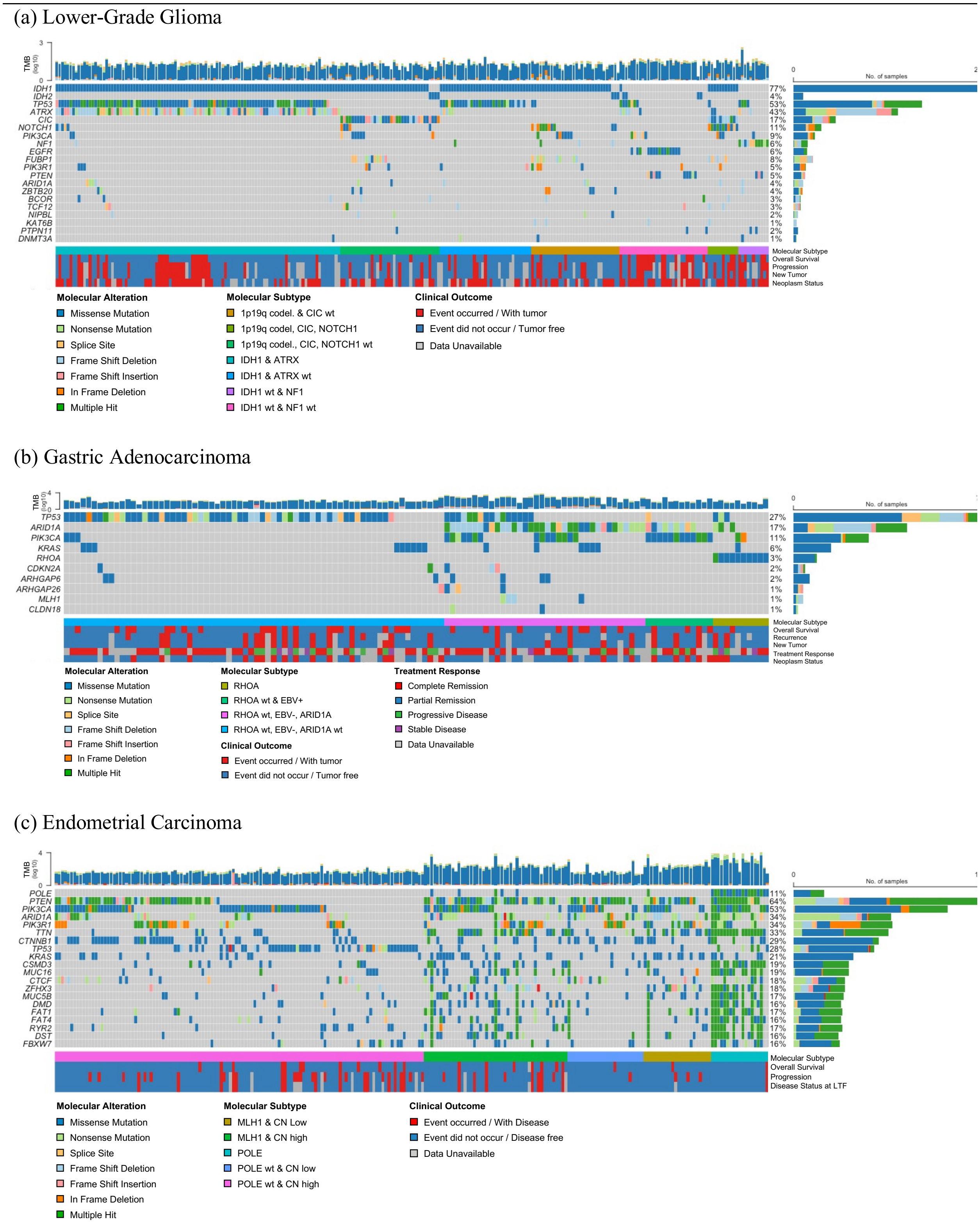

Supplementary Figure 3. Molecular landscape (up to the 20 most frequent somatic alterations), clinical outcomes, and molecular subtypes identified by MuTATE for LGG (a), GA (b), and EC (c)

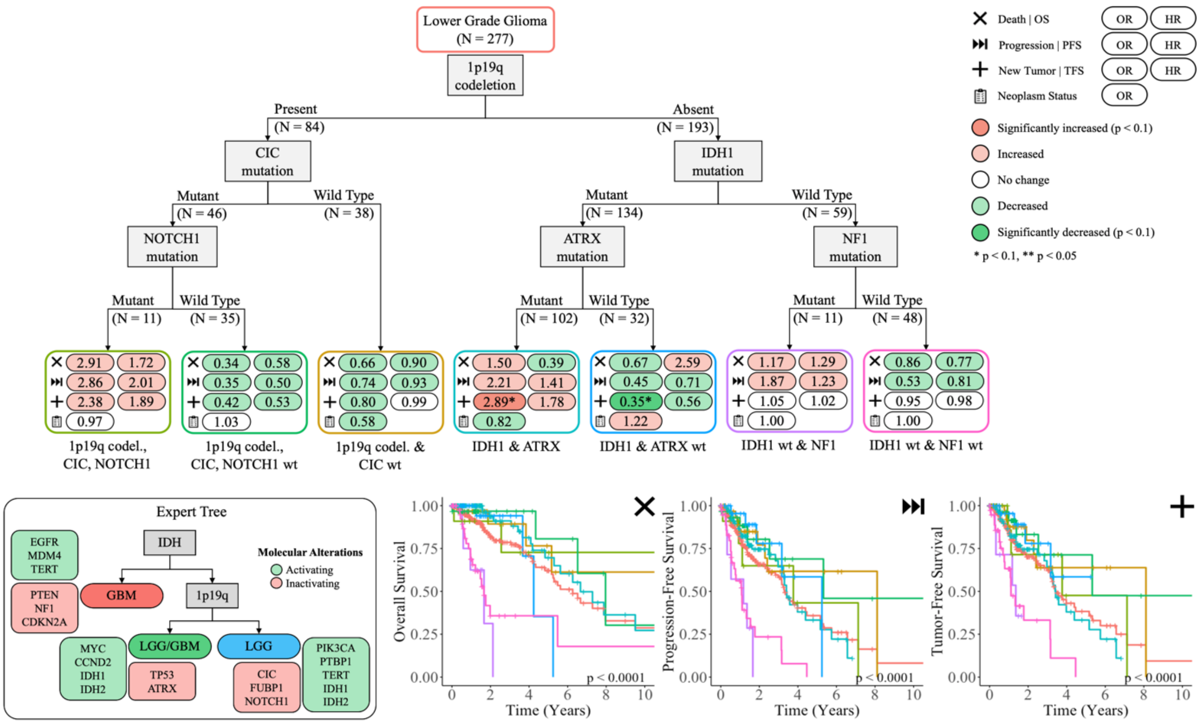

Supplementary Figure 4. MuTATE identifies inactivating alterations, revealing NF1 marker of increased severity in IDH wild-type LGG. MuTATE exceeded published expert-level performance (partitions on IDH mutation and 1p19q codeletion from the expert tree) by automating comprehensive molecular modeling. Partitions (presented in tree nodes) quantified relationships with clinical endpoints, revealing differences in new tumor events in those with or without ATRX mutation in IDH mutant LGG. Multi-target subtypes (presented in leaf nodes) represented significant heterogeneities in survival outcomes shown in Kaplan-Meier curves (overall LGG in orange).

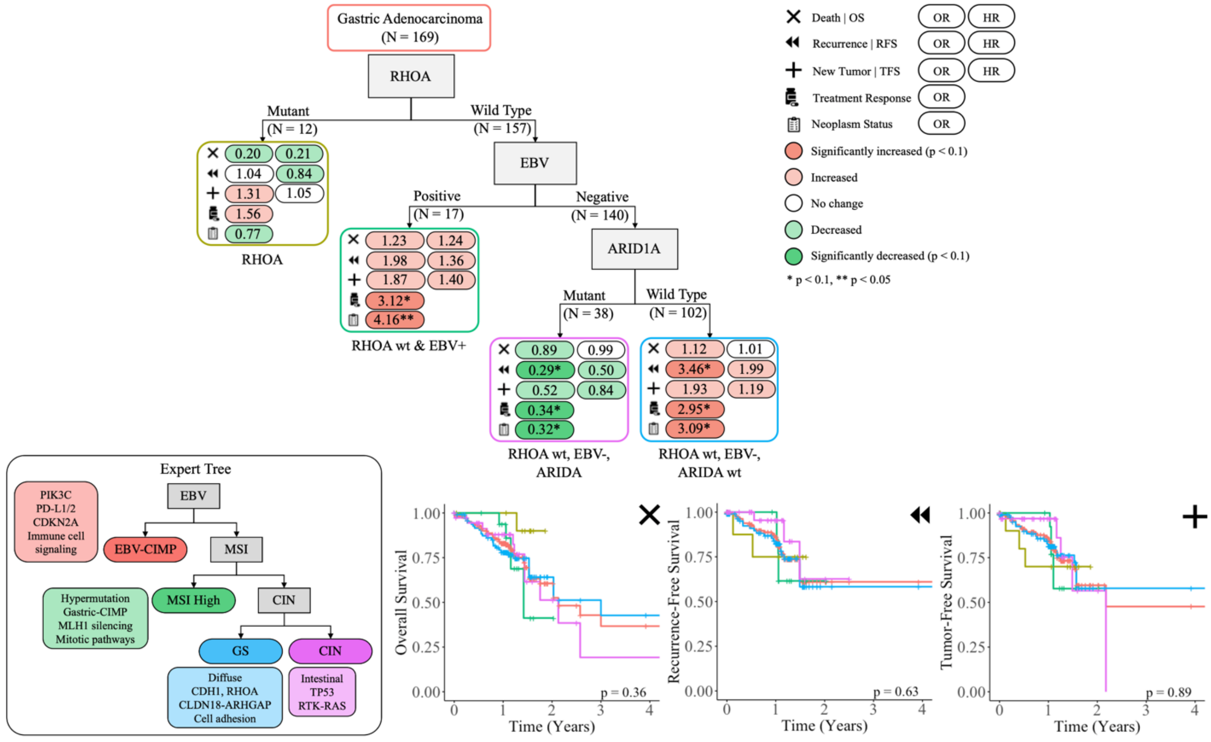

Supplementary Figure 5. MuTATE identifies RHOA and ARID1A, which explain clinical phenotypes and predict treatment response in GA. MuTATE exceeded published expert-level performance (partition on EBV, the expert genomically stable "GS" subtype denoted by RHOA, and the expert chromosomal instability "CIN" subtype denoted by ARID1A) and refined expert architectures by identifying novel partitions on detectable molecular alterations known to affect clinical outcomes in GA. Partitions quantified relationships with clinical endpoints, revealing differences in recurrence, treatment response, and neoplasm status in those with or without ARID1A mutation in RHOA wild type & EBV- GA. Multi-target subtypes represented heterogeneities in clinical outcomes, although significant survival differences were not observed in Kaplan-Meier curves (overall GA in orange).

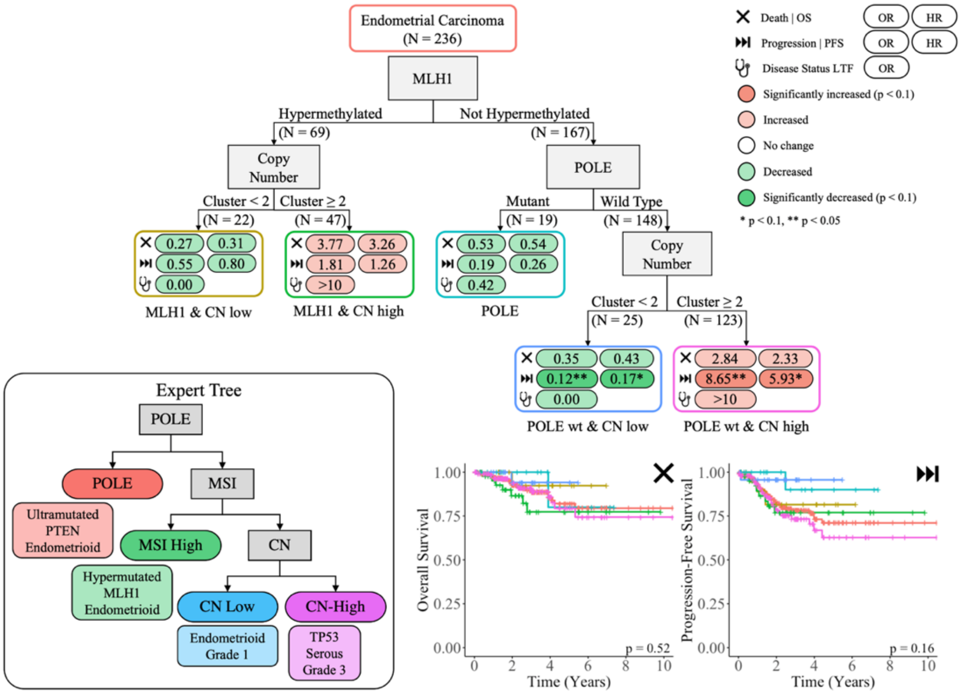

Supplementary Figure 6. MuTATE identifies a novel copy number partition, denoting increased severity, in MLH1 hypermethylated EC. MuTATE exceeded published expert-level performance (partitions on MSI/MHL1, copy number, POLE from the expert tree) and expanded on expert architectures by automating comprehensive molecular modeling. Partitions quantified relationships with clinical endpoints, revealing differences in progression in those with high or low copy number cluster in MLH1 non-hypermethylated & POLE wild type EC. Multi-target subtypes represented heterogeneities in clinical outcomes, although significant survival differences were not observed in Kaplan-Meier curves (overall EC in orange).

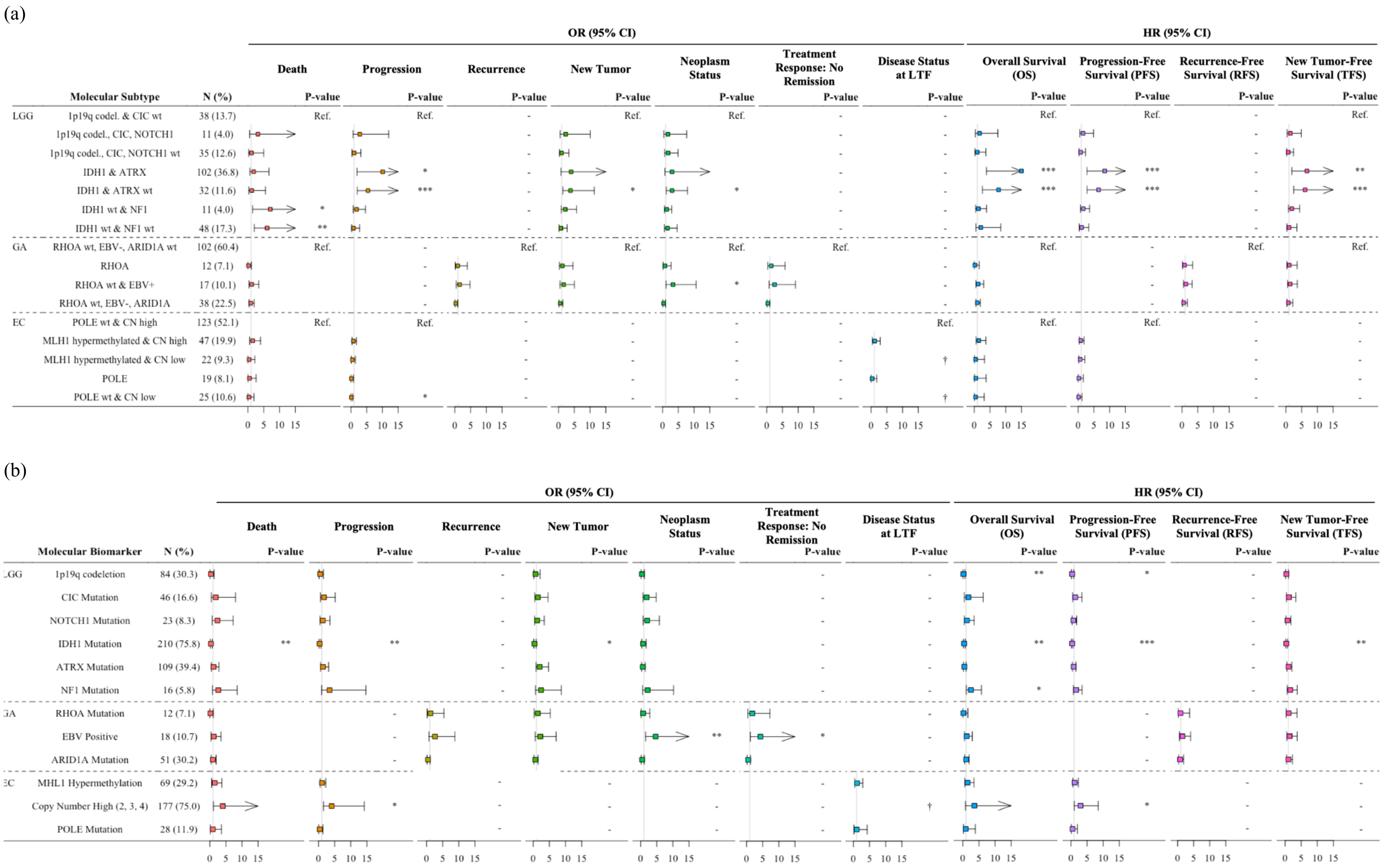

Supplementary Figure 7. Multi-target subtypes reveal clinical phenotype heterogeneities (a) and biomarkers predict progression in multivariable analyses (b). Notably, the IDH & ATRX subtype showed significantly increased risk of death, while IDH & ATRX wild type showed significantly increased risk of progression in LGG. IDH mutation and 1p19q codeletion protected against severe clinical outcomes, while NF1 mutation was associated with increased risk in LGG. EBV+ status indicated significantly increased risk in GA and high copy number group predicted significantly increased risk in EC. Logistic regression was used to obtain multivariable OR (95% CI, P-value) estimates. Cox survival models were used to obtain multivariable HR (95% CI, P-value) estimates. Symbol key: * $p < 0.05$; ** $p < 0.01$; *** $p < 0.001$; † Estimates could not be calculated due to few observed events.
